# Fetal programming effect of rumen-protected methionine on primiparous Angus × Simmental offspring’s performance and skeletal muscle gene expression

**DOI:** 10.1093/jas/skae006

**Published:** 2024-01-10

**Authors:** Gastón F Alfaro, Soren P Rodning, Sonia J Moisá

**Affiliations:** Department of Animal Sciences, Auburn University, Auburn, AL 36849, USA; Department of Animal Sciences, Auburn University, Auburn, AL 36849, USA; Department of Animal Sciences, University of Tennessee, Knoxville, TN 37998, USA

**Keywords:** adipogenesis, beef cattle, fetal programming, gene expression, primiparous dams, rumen-protected methionine

## Abstract

Primiparous Angus × Simmental dams (*n* = 22) with an average body weight (**BW**) of 449 ± 32 kg of BW were divided based on two nutritional treatments: control (**CTRL**) and rumen-protected methionine (**RPM**). The control group received bermudagrass hay, corn gluten, and soybean hulls pellets supplementation (base diet); whereas the RPM group received the base diet in addition to 0.07% of DM of RPM at a fixed rate during the last trimester of gestation and the first ~80 d of lactation, in which calves (*n* = 17) were early weaned. Only male calves were included in this study. After weaning, calves born to RPM dams also received RPM from weaning (day 1) to day 100. Blood sampling and skeletal muscle biopsies for subsequent quantitative polymerase chain reaction (**PCR**) analysis were conducted on days 1, 25, 50, and 100 on calves. Quantitative PCR data were analyzed using GLIMMIX, and blood metabolites concentrations, BW, and body condition score (BCS) were analyzed using the MIXED procedure of SAS. There was no difference in maternal BW and BCS between treatments. Glucose and blood metabolites that served as biomarkers for liver health (e.g., aspartate transaminase, albumin, alkaline phosphatase, and alanine transaminase) were in the normal levels for all calves (*P *> 0.40). Calves in the RPM group had a greater expression of adipogenic genes (e.g., *PPARG*, *LPL*, and *CEBPD*) at day 100 compared with CTRL (*P* < 0.01). In addition, DNA methylation (*DNMT1*) and oxidative stress-related genes (*SOD2* and *NOS3*) in the RPM group were upregulated at day 100 compared with CTRL (*P* < 0.01). These results may suggest that calves born to primiparous dams exposed to RPM supplementation are more prone to develop greater adipose tissue than CTRL calves. Furthermore, RPM supplementation may improve methylation processes, as shown by the upregulation of *DNMT1.* The results shown in our study aim at expanding the knowledge on fetal programming and early-life growth and development of beef cattle under supplementation with RPM.

## Teaser

Supplementation with rumen-protected methionine alters the expression of genes related to adipogenesis, DNA methylation, and oxidative stress in beef male calves.

## Introduction

The utilization of protected amino acids has been rising in the dairy industry during the last decades. High-producing dairy cows are usually deficient in methionine, and dietary supplementation improves overall health and milk production (Rulquin and Delaby, 1997; Kowalski et al., 2003; Misciatteilli et al., 2003). Nevertheless, the benefits of maternal supplementation with rumen-protected methionine (**RPM**) also extend into the gestating offspring. In addition, methionine plays an essential role in methylation because it is a precursor for *S*-adenosyl methionine, known as the major methyl-donor compound (Cantoni, 1975). Due to the positive results obtained in investigation on dairy cattle, a novel area of research analyzes the effects of RPM supplementation on beef cattle.

In the Southeast region of the United States, the cow–calf operation system represents the predominant method of beef production. In addition, beef quality determines the price of beef at the consumer level in the United States. The main driver of beef quality is intramuscular fat or marbling, which is developed early in life in ruminants (Du et al., 2015; Drouillard, 2018).

Even though methionine is not usually a limiting amino acid in beef cattle, its effect on DNA methylation that could enhance animal performance is worthy of investigation. Thus, identifying novel, beneficial dietary strategies for improving both the dam and the offspring performance could result in positive productive and economic outcomes in cow–calf systems.

To the best of our knowledge, no previous evidence showed the effects of maternal RPM supplementation on beef cattle primiparous dams on adipogenic, oxidative stress, and DNA methylation on offspring’s skeletal muscle. We hypothesized that supplementing with RPM to both, the dam during the last trimester of gestation and lactation, and the offspring after weaning, would improve animal performance and targeted gene expression. Therefore, the objectives of our study were (1) to identify changes in body weight (**BW**), body condition score (**BCS**), and milk production on dams receiving RPM during the last trimester of gestation and the first ~80 d of lactation; (2) to evaluate changes in BW, average daily gain (**ADG**), and blood metabolites related to liver health on calves supplemented with RPM; and (3) to analyze changes in adipogenic, oxidative stress, and DNA methylation-related genes of calves born to dams supplemented with RPM.

## Materials and Methods

All procedures were approved by the Auburn University Animal Care and Use Committee (PRN# 2017-3154). This study was conducted from October 2017 to August 2018.

### Animals, dietary treatments, and experimental design

**Figure 1.**
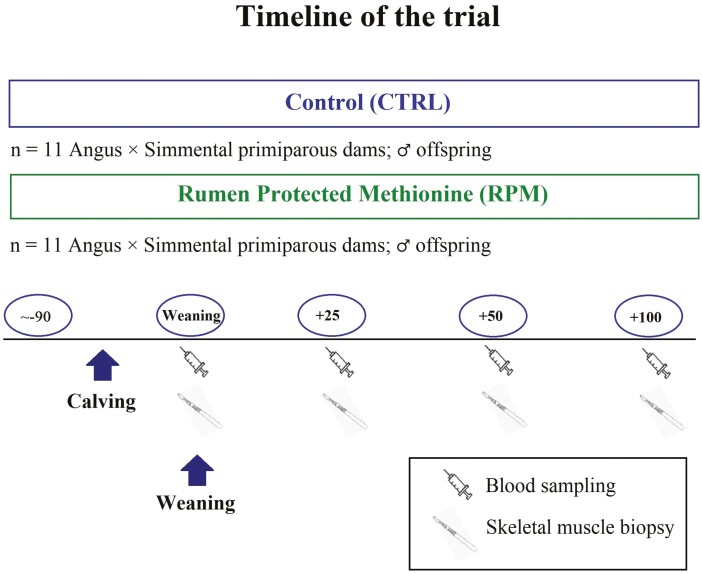
Experimental design and timeline of the study.

A group of pregnant, primiparous Angus, Angus × Simmental, and Simmental dams (*n* = 22) with an average BW (449 ± 32 kg) were located at North Auburn Research Unit, Auburn University, AL for all the experimental procedures (32°41ʹN, 85°30ʹW; Figure 1). Primiparous dams were artificially inseminated with semen from a mature, purebred Simmental bull. Pregnancy checking was performed before the beginning of the study to confirm the pregnancy status. Calves’ sex was identified during pregnancy checking using trans-rectal ultrasound. Calving season ranged from January to March 2018. In addition, primiparous dams were randomly split into two treatment groups based on RPM inclusion in the diet: RPM treatment (*n* = 11) and Control treatment (**CTRL**; *n* = 11). A successful adaptation to individual feeders separated through fences used for better control of individual administration and intake of nutritional treatments was performed in 5 d. After the adaptation period, dams received bermudagrass (*Cynodon dactylon*) hay ad libitum in combination with an individual, daily supplementation of 2.06 kg soybean hulls and corn gluten pellets in a 1:1 ratio (Table 1). In addition, RPM dams received 8 g/head/d (i.e., 0.07% of DM) of top-dressed RPM at a fixed rate (Smartamine, Adisseo NA, Alpharetta, GA). Smartamine presents a minimum of 70% of Met, coated with a pH-sensitive polymer with a Met bioavailability of 80% (Schwab, 2007). Therefore, per 8 g of RPM, animals received a minimum of 4.48 g of metabolizable Met (Alfaro et al., 2020). Dams were supplemented with RPM during the last trimester of gestation (~90 d) and during the first ~80 d of lactation. Control dams did not receive RPM supplementation during these time periods. BCS was visually estimated by the same trained person on each dam at calving and 60 d after calving. Body condition was scored in increments of 1 unit, using a scale ranging from 1 to 9 (1 = thin and 9 = fat; Renquist et al., 2006).

**Table 1.** Chemical composition of maternal base diets fed to dams during the peripartal period

Diet fed to primiparous dams						
Ingredient^1^	DM, %AF	CP, %DM	NDF, %DM	ADF, %DM	TDN, %DM	Crude fat, %DM
Bermudagrass hay	84.7	8.1	75.66	42.89	53.65	–
Soybean hull/corn gluten supplement	90.84	16.23	47	27.86	90.25	11.82

Bermudagrass hay was fed ad libitum, whereas soybean meal and corn gluten supplement was offered in a rate of 2.72 kg/hd/d.

^1^All ingredients are expressed on a DM basis.

Abbreviations: DM, dry matter; CP, crude protein; NDF, neutral detergent fiber; ADF, acid detergent fiber; TDN, total digestible nutrients.

At calving, birth date and BW of calves were recorded only in male calves, whereas female calves were not included in the study. The total of male calves used for our study was eight for CTRL group, and nine for RPM group. Calves had free access to maternal milk, bermudagrass hay, and creep feeder starter (Rough-N-Ready, Archer Daniels Midland Company, Quincy, IL) to stimulate solid feed intake (Table 2). Calves were at least 45 d old at weaning. After early weaning (80 d of life on average), calves were relocated to Sugg Laboratory for Animal Health Research pens (32°36ʹN, 85°31ʹW), College of Veterinary Medicine, Auburn, AL, where they had ad libitum access to bermudagrass hay in addition to 1% of BW of early weaning feed. Furthermore, calves born to RPM dams continued their maternal nutritional treatment postweaning (Tables 3 and 4). The inclusion rate of RPM in calves was adjusted after each BW measurement, and it ranged from 1.5 to 3 g/hd/d (0.07% dry matter intake [DMI]). The supplementation with RPM to calves was top-dressed to early weaning feed after successful adaptation to individual feeders separated throughout fences.

**Table 2.** Chemical composition of offspring base diets fed to calves during the lactation period

Ingredient ^1^	DM, %AF	CP, %DM	NDF. %DM	ADF, %DM	TDN, %DM	Crude fat, %DM
Bermudagrass hay	84.70	8.10	75.66	42.89	53.65	–
Creep Feed Starter^2^	89.53	21.24	19.34	11.27	99.47	7.72

Calves had ad libitum access to maternal milk, bermudagrass hay, and creep feed starter.

^1^All ingredients are expressed in a DM basis.

^2^Rough “N” Ready, ADM, Chicago, IL.

Abbreviations: DM, dry matter; CP, crude protein; NDF, neutral detergent fiber; ADF, acid detergent fiber; TDN, total digestible nutrients.

**Table 3.** Chemical composition of offspring base diets fed to calves during the early weaning period

Chemical composition of early-wean feed	
Ingredients ^1^	% DM
Distiller grain	12.5
Limestone 38%	0.5
Corn	17.5
Cotton seed hulls, dry	10
Soybean hulls, dry	43.4
Molasses	5
Soybean meal, dry	10
Rumensin 80^2^	0.02
Salt	0.5
Availa 4^3^	0.1
J&R all purpose^4^ mineral	0.5
Total	100

^1^All ingredients are expressed in a DM basis.

^2^Elanco Animal Health, Greenfield, IN.

^3^Zinpro Corporation, Eden Prairie, MN.

^4^Z&R Supply Inc., East Dubuque, IL.

**Table 4.** Diet fed to calves born to primiparous dams

Ingredients ^1^	DM, % AF	CP, % DM	NDF, %DM	ADF, %DM	TDN, %DM	Crude fat, %DM
Bermudagrass hay	92.35	8.27	71.61	39.34	57.70	–
Early-wean feed	89.30	15.93	37.93	26.24	68.26	4.22

Calves were fed ad libitum access to bermudagrass hay and 1% of BW as DM basis of early wean feed.

^1^All ingredients are expressed in a DM basis.

Abbreviations: DM, dry matter; CP, crude protein; NDF, neutral detergent fiber; ADF, acid detergent fiber; TDN, total digestible nutrients.

Both primiparous dams and calves had ad libitum access to mineral salt supplement Stockman’s complete (Sweetlix, Mankato, MN) containing salt (NaCl, 11% to 13%), Ca (23% to 27.5%), Mg (4%, min), Mn (3,950 ppm, min), Cu (2,500 ppm, min), Zn (6,000 ppm, min), I (150 ppm, min), Se (26 ppm, min), Co (40 ppm), Vit A (660,000 IU/kg, min), Vit D3 (66,000 IU/kg, min), and Vit E (440 IU/kg, ppm).

Body weight from dams was obtained on October 1, 2017 (beginning of the study) and on March 5, 2018 (weaning). Calves’ BW was obtained at weaning, 25, 50, 80, and 100 d after weaning. Furthermore, a traditional knife castration was performed 35 d after weaning. Immediately after castration, calves were implanted with Synovex C (Zoetis, Parsippany, NJ), containing 100 mg progesterone and 10 mg estradiol benzoate. Simultaneously, calves received vaccination with Vision7 (Merck, Kenilworth, NJ). Finally, at 84 d after weaning, calves were orally dewormed using a dewormer suspension 10% (Safe-Guard, Kenilworth, NJ), which contains fenbendazole as the main active ingredient, at the recommended rate of 5 mL/100 kg.

### Skeletal muscle biopsies

Biopsies of *longissimus dorsi* skeletal muscle were performed at weaning, 25, 50, and 100 d after weaning for gene expression analysis using real-time quantitative polymerase chain reaction (**qPCR**). Each muscle sample was obtained from the left side with the incision side positioned progressively more caudal with each sequential biopsy separated for at least 5 cm. Anesthesia injections of 5 mL of lidocaine 2% (VetOne, Boise, ID) were injected before each biopsy incision. A biopsy core of muscle (600 to 800 mg) was removed using a sterile skeletal muscle biopsy needle (Bard Magnum Biopsy Needle 12 Gauge 16 cm Ultra Sharp Tip—N1216, Covington, GA), placed in sterile 2 mL cryovial tubes, and immediately stored in liquid nitrogen for transportation and storage at −80 °C until further analysis. No signs of infection, swelling, or external bleeding were detected on the biopsy site after each procedure or in the following days. Biopsy procedures did not affect feed and water intake during the consequent days (Alfaro et al., 2020).

### Serum analyses

Ten milliliters of whole blood was collected via jugular venipuncture on calves at 1 (weaning), 25, 50, and 100 d after weaning into blood collection tubes (BD Vacutainer, Becton Dickinson, Franklin Lakes, NJ). Serum was separated by centrifugation at 1,500 × *g* for 15 min, and an aliquot was stored frozen at −20 °C until analyzed for glucose, albumin, aspartate transaminase (**AST**), alanine transaminase (**ALT**), and alkaline phosphatase (**ALP**) at the Auburn University Endocrine Diagnostic Lab, Auburn, AL. All serum analyses were performed using a Roche/Hitachi Cobas C c 311 analyzer (Roche, Basel, Switzerland) for clinical chemistry (Bowling and Katayev, 2010).

### RNA extraction

Approximately 100 mg of skeletal muscle tissue from each calf at each biopsy date was immersed in 1 mL of QIAzol Lysis Reagent (Qiagen, Hilden, Germany; Cat. #: 79306), homogenized for 1 min, cooled on ice for 1 min, and finally homogenized for another extra 1 min. After 5 min of incubation on ice, each sample received 0.2 mL of chloroform and was vigorously shaken by hand for 15 s. Then, after 2 min of incubation, samples were centrifuged for 10 min at 12,000 × *g* at 4 °C. After centrifugation, each tube presented a lower red phenol–chloroform layer, interphase, and a colorless upper aqueous phase, which contains the RNA. Consequently, the upper phase was transferred to a new tube, and 0.5 mL of isopropanol was added and then incubated for 10 min. The following step consisted of centrifugation for 10 min at 12,000 × *g* at 4 °C in which total RNA is precipitated as a white pellet at the bottom of the tube. The supernatant was discarded, and the pellet was resuspended in 1 mL of 75% ethanol and centrifuged for 5 min at 7,500 × *g* at 4 °C. Afterward, the tubes containing RNA pellets were opened for 10 min under the hood in order to be exposed to air drying. Finally, 20 µL of RNAase-free water was added directly to resuspend the RNA pellet. The RNA concentration was obtained using Nanodrop (Nanodrop One^C^, ThermoFisher, Waltham, MA). Samples were cleaned using RNA Clean & Concentrator kit according to the manufacturer’s protocol (Zymo Research Catalog # R1015). The integrity of RNA was assessed by electrophoresis gel, and samples showing two clear, parallel ribosomal bands were considered acceptable.

### Primer design

The complementary DNA (**cDNA**) sequences for genes used were found at National Center for Biotechnology Information (**NCBI**; https://www.ncbi.nlm.nih.gov/) or University of California-Santa Cruz’s Genome Browser (https://www.genome.ucsc.edu/). The sequences obtained were entered into Primer Express 3.0.1 software (ABI). The default settings (TaqMan MGB quantification) were used; however, amplicon size was modified to 100 bp (Supplementary Table S1). The designed primer sequences were uploaded in the blast tool of NCBI Nucleotide Blast (Supplementary Table S2) and ordered from Integrated DNA Technologies (https://www.idtdna.com) to confirm that the designed sequences belong to the gene of interest (Supplementary Table S3).

### cDNA synthesis

cDNA was transcribed from clean RNA at a concentration of 100 ng/µL. First, Master Mix 1 (**MM1**) was prepared by mixing 9 µL of RNase-free water with 1 µL of Random Primers (Roche Diagnostics, Indianapolis, IN). Later, 1 µL of 100 ng total RNA was added. The mixture was incubated at 65 °C for 5 min. Then, the samples were incubated on ice for 3 min. For each sample, MM2 was prepared by mixing 1.625 µL RNase-free water, 4 µL 5× first-strand buffer, 1 µL Oligo dT18, 2 µL 10 mM dNTP mix (10 mM), 0.25 µL of revert aid (200 U/µL), and 0.125 µL of RNase inhibitor (20 U/µL). Then, MM2 was mixed with MM1 + RNA (final volume of 20 µL per reaction). The incubation protocol was as follows: 25 °C for 5 min, 42 °C for 60 min, and 70 °C for 5 min followed by 4 °C. A pooled sample was obtained from all samples to design the standard curve. Then, the pooled sample was diluted to a 1:2 ratio with RNase-free water for the first standard curve point. The subsequent standard curve points were diluted to a 1:4 ratio.

### Preliminary primer testing

The testing of primers was performed as follows: 1 µL of forward primer, 1 µL reverse primer, 8 µL of pooled cDNA, and 10 µL of Perfecta SYBR Green through PCR. Samples were placed in a gradient thermocycler (Eppendorf nexus, Hamburg, Germany) for 2 min at 50 °C, 10 min at 95 °C, 40 cycles of 15 s each at 95 °C and 1 min at 60 °C for denaturation. Five microliters of the PCR product were transferred to a new 0.2-mL PCR tube for agarose gel electrophoresis analysis and mixed with 2 µL of loading dye. The ladder was prepared by mixing 0.6 µL of ladder (25 bp, Invitrogen, Carlsbad, CA) with 2 µL of loading dye. In addition, 3 g of agarose (OmniPur, Calbiochem, San Diego, CA) was dissolved in 150 mL of TAE Buffer (Invitrogen; Cat # 15558-026). The agarose mix was heated for 1 min in a microwave or until completely dissolved. Two microliters of SYBR Safe were added to the agarose mix before cooling and then placed in the agarose gel apparatus. Ladders were added to the first well of each row, and the samples were added to the remaining empty wells. The gel ran at 80 mV until samples and ladder reached three-quarters of the gel path.

Bio-Rad Chemi Doc XRS+ (Bio-Rad, Hercules, CA) apparatus was used to analyze the gel, utilizing Image Lab software (Bio-Rad). The accepted primers had a clear and single band at 100 bp. Finally, the accepted PCR products were cleaned with a purification kit (QIAquick PCR Purification Kit Qiagen; Cat. # 28104), before sending for Sanger sequencing analysis to the University of Illinois Core Sequencing facility. Sequencing results were blasted in the NCBI website. The sequencing results that matched the primers blast were utilized. The following genes were selected as housekeeping or internal controls: mitochondrial ribosome-associated GTPase 1 (***MTG1***), ribosomal protein S15a (***RPS15A***), and ubiquitously expressed prefoldin-like chaperone (***UXT***). Furthermore, peroxisome proliferator-activated receptor gamma (***PPARg***), lipoprotein lipase **(*LPL*)**, CCAAT/enhancer binding protein (**CEBP**) gamma (***CEBPG***), CEBP delta (***CEBPD***), DNA methyltransferase 1 (***DNMT1***), and superoxide dismutase 2 (***SOD2***) were the selected target genes for this study.

### Real-time polymerase chain reaction

Eight microliters of diluted cDNA sample, negative control, and standard curve were pipetted into their respective wells of a 96-well reaction plate (MicroAmp Optical, ThermoFisher) in duplicates. Later, 10 µL of SYBR Green Master Mix, composed of 8 µL of PerfeCTa SYBR Green (Quanta Biosciences INC., Beverly, MA), 0.6 µL forward primer, 0.6 µL reverse primer, and 0.4 µL of water, was pipetted into each well. The PCR reaction was executed in an ABI Prism 7500 HT SDS machine set to 2 min at 50 °C, 10 min at 95 °C for holding stage; 40 cycles of 15 s each at 95 °C and 1 min at 60 °C for cycling stage; and 15 s at 95 °C, 1 min at 60 °C, 30 s at 95 °C, and 15 s at 60 °C for dissociation curve stage (Supplementary Table S4). The data obtained were analyzed using the 7500 HT Sequence Detection Systems Software (version 2.3, Applied Biosystems, Foster City, CA). Prior to statistical analysis, qPCR data were normalized using the geometric mean of housekeeping genes (*UXT*, *MTG1*, and *RPS15A*).

### Statistical analysis

The response variables analyzed included qPCR data, BW, BCS, and blood metabolites at different time points. Quantitative PCR data were analyzed using the GLIMMIX procedure of SAS (SAS 9.4 Institute, Cary, NC). Statistical analyses were performed using Ct values of targeted genes, whereas graphs were designed based on quantity values (2^∆∆Ct^). Blood metabolites concentrations, BW, and BCS were analyzed using the MIXED procedure of SAS. Time and treatment were considered the fixed effect in the statistical model. The random effect in all models was animal within treatment. The MIXED procedure of SAS included a repeated-measure statement with an unstructured covariate structure.

Fixed effects in the statistical model for each variable analyzed (i.e., genes and blood metabolites) included treatment (RPM or CTRL), time (days 1, 25, 50, and 100) on experiment, and treatment × time on experiment interaction when appropriate (e.g., mRNA expression over time). Statistically significant differences were declared at *P* < 0.05 and tendencies at *P *> 0.05 and < 0.10. The statistical model used was Y_ijl_ = μ + C_i_ + T_j_ + S_l_ + (C × T)_ij_ + ε_ijl_, where, Y_ijl_ is the background-adjusted normalized parameter value (i.e., Ct or blood data value); μ is the overall mean; C_i_ is the fixed effect of time (4 or 5 levels); T_j_ is the fixed effect of treatment (2 levels); S_l_ is the random effect of heifer nested within treatment; C × T is the interactions of time by treatment, and ε_ijl_ is the random error (0, ${\sigma_{e}}^{2}$) associated with Y_ijl_.

## Results

### Animal performance

**Figure 2.**
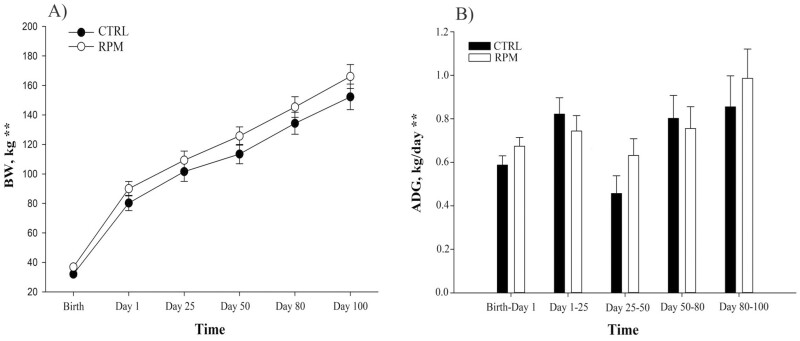
Effect of in utero, lactation, and postweaning exposure to methionine in calves born to primiparous dams receiving RPM during the peripartal period, on (A) BW and (B) ADG. Statistically significant differences were declared at *P* ≤ 0.05 and tendencies at *P* > 0.05 and < 0.1. **Tme effect. Error bars represent SEM.

Body weight did not have a treatment × time interaction (*P *= 0.885; Table 5), although it presented a time effect (*P *< 0.001). At the beginning of the study, dams in RPM had a BW of 454 ± 32 kg and their BW decreased to 376 ± 41 kg at weaning; whereas CTRL dams had an initial BW of 442 ± 33 kg which decreased to 362 ± 41 kg at weaning. The inclusion of RPM did not affect BW in dams (*P *= 0.445; Figure 2A).

**Table 5.** Effect of RPM supplementation during the last trimester of gestation and lactation on BW (kg) and BCS of primiparous dams

Item	Treatment		SEM	*P-*value		
	CTRL	RPM		Trt.	Time	Trt. × Time
BW, kg						
Initial BW	442.33^a^	454.04^a^	28.76	0.445	0.001	0.885
BW weaning	362.73^b^	376.46^b^				
BCS						
BCS calving	5.63^a^	5.44^a^	0.22	0.769	0.001	0.457
BCS 60 d post-calving	4.88^b^	4.89^b^				

Statistical differences were declared at *P ≤* 0.05, and tendencies at *P *> 0.05 and < 0.1.

Table shows *P*-values for treatment effect (Trt.), time effect (Time), and treatment × time interaction (Trt. × Time).

Superscript letters represent statistical differences between two time points for the same treatment.

There was no treatment × time interaction (*P* = 0.457; Table 5) in BCS, however, there was a slight reduction in dam’s BCS, represented by a time effect (*P *< 0.001). BCS decreased from 5.44 ± 0.52 to 4.89 ± 0.62 in RPM, and 5.63 ± 0.52 to 4.88 ± 0.62 in CTRL dams from calving to 60 d after calving. Supplementation with RPM did not affect BCS (*P* = 0.769; Figure 2B).

Calves born to primiparous dams did not present a significant treatment × time interaction in BW (*P *= 0.218) or ADG (*P = *0.562; Figure 2). Similarly, neither maternal nor offspring supplementation with methionine impacted calves’ BW (treatment effect, *P *= 0.176). However, BW of calves increased significantly in the first 100 d after weaning showing a time effect (*P *< 0.001).

### Blood analyses

**Figure 3.**
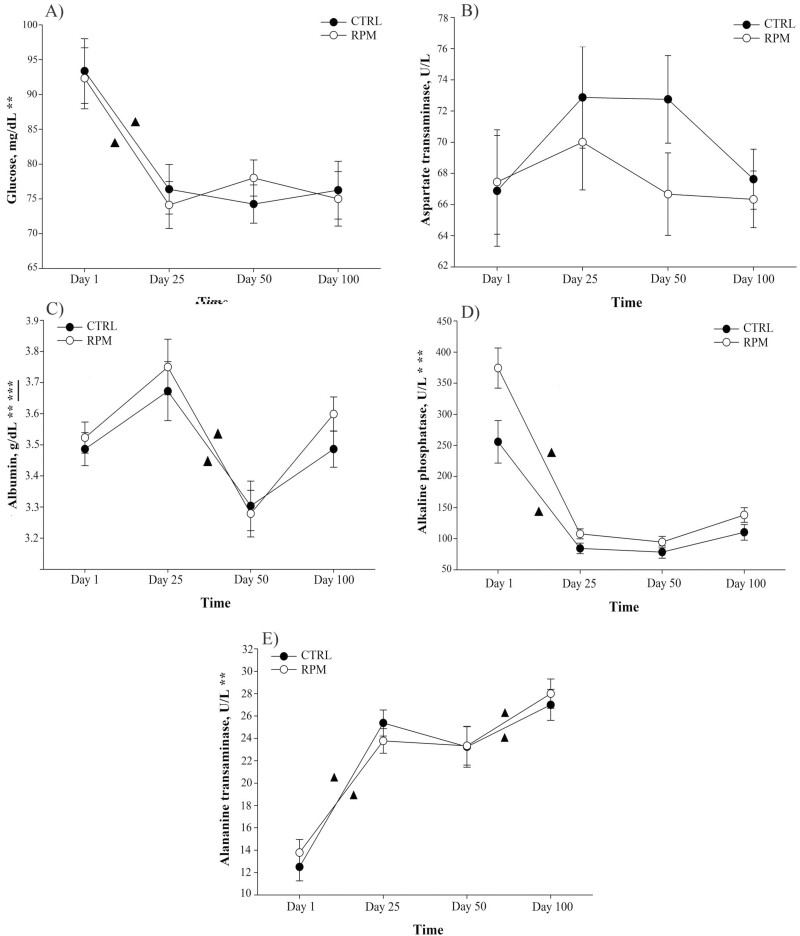
Effect of in utero, lactation, and postweaning exposure to methionine in calves born to primiparous dams receiving RPM supplementation during the peripartal period on blood metabolites. (A) Glucose, (B) AST, (C) albumin, (D) ALP, and (E) ALT. Statistically significant differences were declared at *P* ≤ 0.05 and tendencies at *P* > 0.05 and < 0.1. ***Treatment × time interaction, **time effect, and *treatment effect. Tendencies are denoted if symbols (*, **, or ***) are underlined. Symbols (▲) on lines denote significant differences (*P* < 0.05) between two time points for the same treatment, and symbols (●) denote significant differences (*P* < 0.05) between treatments at the same time point. Error bars represent SEM.

Plasma glucose concentration did not have a treatment × time interaction (*P* = 0.412). Although, its serum levels decreased significantly from days 1 to 25, from 92.3 ± 13.1 to 74.1 ± 10.1 mg/dL and 93.4 ± 4.6 to 76.4 ± 3.5 mg/dL in RPM and CTRL groups, respectively (*P* < 0.001; Figure 3A). Plasma glucose concentration remained stable from days 25 to 100 after weaning. However, supplementation with methionine did not affect circulating glucose (*P *= 0.962).

There was no treatment × time interaction (*P* = 0.494), treatment effect (*P *= 0.406), or time effect (*P *= 0.154) for AST concentration in serum (Figure 3B). Furthermore, there was a tendency for treatment × time interaction in plasma albumin (*P *= 0.082; Figure 3C). Albumin concentration decreased from days 25 to 50 from 3.75 ± 0.27 to 3.27 ± 0.22 g/dL in the RPM group and 3.67 ± 0.27 to 3.30 ± 0.22 g/dL in CTRL group (*P *< 0.001). However, there was no difference between treatment groups (*P *= 0.490).

There was no treatment × time interaction for serum ALP concentration (*P *= 0.553). Although, plasma ALP decreased significantly from days 1 to 25 from 374.4 ± 96.5 to 107.8 ± 24.0 U/L in RPM, and 255.7 ± 96.5 to 84.4 ± 24.0 U/L in CTRL group (*P *< 0.001; Figure 3D).

Finally, there was no treatment effect × time interaction (*P* = 0.89) or treatment effect (*P* = 0.323). However, ALT increased markedly from days 1 to 25 from 13.8 ± 3.5 to 23.8 ± 3.3 U/L in RPM and 12.5 ± 3.5 to 25.37 ± 3.3 U/L in CTRL group (Figure 3E). Similarly, the ALT concentration increased from days 50 to 100 from 23.3 ± 5.2 to 28.0 ± 3.9 U/L in RPM and 23.2 ± 5.1 to 27.0 ± 3.9 U/L in CTRL group (*P* < 0.001).

### Gene expression

**Figure 4.**
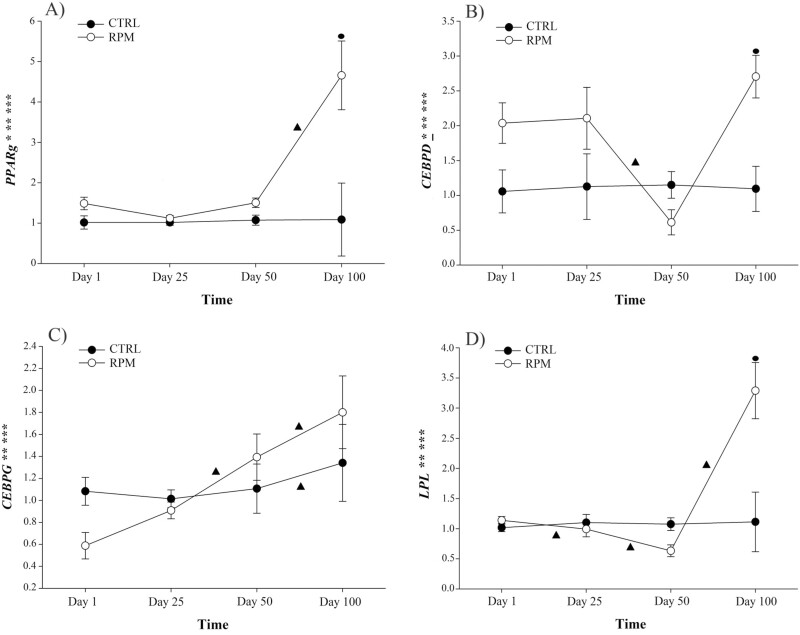
Effect of in utero, lactation, and postweaning exposure to methionine in calves born to primiparous dams receiving RPM supplementation during the peripartal period on adipogenic gene network (fold change). A) *PPARG*, B) *CEBPD*, C) *CEBPG* and D) *LPL*. Statistically significant differences were declared at *P* ≤ 0.05 and tendencies at *P* > 0.05 and < 0.1. ***Treatment × time interaction, **time effect, and *treatment effect. Tendencies are denoted if symbols (*, **, or ***) are underlined. Symbols (▲) on lines denote significant differences (*P* < 0.05) between two time points for the same treatment. Errors bars represent SEM.

There was a treatment × time interaction (*P *< 0.001), treatment effect (*P *< 0.001), and time effect (*P *< 0.001) for *PPARg* expression (Figure 4; Table 6). Only in RPM group, it was upregulated from days 50 to 100. Furthermore, the *PPARg* expression of RPM group at day 100 was markedly greater as compared to CTRL group (*P *= 0.003).

**Table 6.** Overall least mean squares fold change values for expression of genes analyzed in *longissimus dorsi* muscle of calves born to primiparous dams in CTRL and RPM groups

Gene transcript	Day 1		Day 25		Day 50		Day 100		SEM	*P-*value		
	CTRL	RPM	CTRL	RPM	CTRL	RPM	CTRL	RPM		Trt.	Time	Trt. × Time
*PPARG*	1.0162	1.4869	1.0178	1.1206	1.0734	1.5037	1.0873	4.6592	0.9015	0.001	0.001	0.001
*LPL*	1.0171	1.1382	1.1006	0.9925	1.0753	0.6329	1.1122	3.2893	0.4945	0.178	0.001	0.001
*CEBPG*	1.0827	0.5881	1.0139	0.9083	1.1068	1.3933	1.3411	1.8002	0.3503	0.965	0.001	0.010
*CEBPD*	1.0567	2.0379	1.1266	2.1079	1.1498	0.6127	1.094	2.705	0.471	0.078	0.001	0.016
*DNMT1*	1.007	0.9351	1.0041	0.9978	1.044	1.582	1.1158	1.871	0.1587	0.004	0.001	0.001
*SOD2*	1.0075	0.9125	1.0062	0.9269	1.0474	0.5917	1.1516	2.038	0.1796	0.875	0.001	0.003
*NOS3*	1.0359	0.8697	1.0103	1.3847	1.0757	1.0018	1.2065	2.5812	0.2564	0.012	0.001	0.001

Statistical differences were declared at *P ≤* 0.05, and tendencies at *P *> 0.05 and < 0.1.

Table shows *P*-values for treatment effect (Trt.), time effect (Time), and treatment × time interaction (Trt. × Time).

There was a treatment × time interaction (*P *= 0.016) and time effect (*P* < 0.001), and a treatment tendency (*P *= 0.078) for *CEBPD* expression (Figure 4B). In the RPM group, there was a downregulation from days 25 to 50. At day 100, RPM group had a greater *CEBPD* expression as compared CTRL group. Furthermore, both *CEBPG* and *LPL* expression showed a treatment × time interaction (*P *< 0.001 and *P *= 0.010, respectively) and time effect (*P *< 0.001; Figure 4C and D, respectively). There was a decrease in the expression of *LPL* in RPM group from days 1 to 25 and from days 25 to 50 (*P *< 0.05); however, there was a significant increase from days 50 to 100. At day 100, RPM group showed greater *LPL* expression as compared to CTRL group. Expression of *CEBPG* was upregulated in RPM group between days 25 to 50, and days 50 to 100. Similarly, CTRL group had upregulation between days 50 to 100.

**Figure 5.**
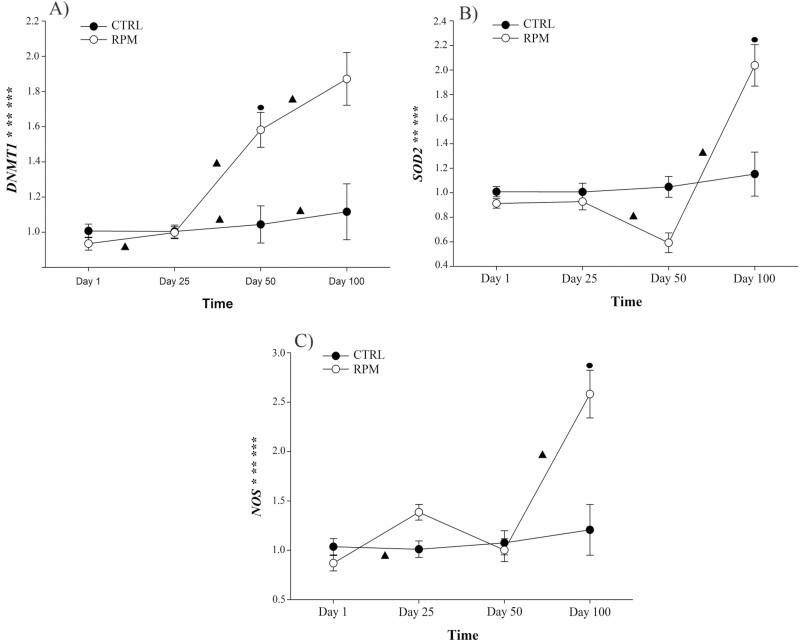
Effect of in utero, lactation, and postweaning exposure to methionine in calves born to primiparous dams receiving RPM supplementation during the peripartal period on DNA methylation and oxidative stress-related genes (fold change). A) *DNMT1*, B) *SOD2*, C) *NOS*. Statistically significant differences were declared at *P* ≤ 0.05 and tendencies at *P* > 0.05 and < 0.1. ***Treatment × time interaction, **time effect, and *treatment effect. Tendencies are denoted if symbols (*, **, or ***) are underlined. Symbols (▲) on lines denote significant differences (*P* < 0.05) between two time points for the same treatment, and symbols (●) denote significant differences (*P* < 0.05) between treatments at the same time point. Error bars represent SEM.

Finally, there was a treatment × time interaction (*P* < 0.01) and a time effect (*P* < 0.001) for *DNMT1, SOD2,* and, *NOS3* expression (Figure 5A–C, respectively). Furthermore, there was a treatment effect in *DNMT1* and *NOS3* expression (*P* < 0.004 and *P* = 0.011, respectively). At day 100, calves in the RPM group had greater *SOD2* expression (Figure 5B). There was downregulation of *NOS3* in CTRL group between days 1 and 25; whereas there was an upregulation of *NOS3* in RPM group from days 50 to 100 (Figure 5C). At day 100, calves in RPM group had a greater expression of *NOS3* compared with those in CTRL calves (Figure 5C). Furthermore, there was an upregulation of *DNMT1* in RPM group from days 1 to 25, days 25 to 50, and days 50 to 100 (Figure 5A). Similarly, calves in CTRL group also showed an upregulation of *DNMT1* from days 25 to 50, and days 50 to 100. Lastly, RPM group had a downregulation of *SOD2* expression between days 25 to 50, and upregulation from days 50 to 100 (Figure 5B).

## Discussion

### Animal performance

#### Maternal BW and BCS

The peripartal period is characterized by a reduction in maternal BW. During this period, ruminal capacity decreases due to the exponential growing of the fetus during the last trimester of gestation. Therefore, DMI decreases while approaching calving (Stanley et al., 1993). Interestingly, as reported by Redifer et al. (2021), calf BW represents ~6% of Angus × Simmental primiparous dams BW at prepartum. The same study showed that, in addition to the fetus’ weight, the placenta represents a greater percentage of BW in primiparous compared with mature dams. Therefore, the decrease of maternal BW from the last trimester of gestation to calving could be affected by the BW of the fetus and the placenta.

BCS is a visual estimation utilized by producers that indicates animal’s body energy reserves and serves as a valuable tool to identify the overall nutritional status of the animals. Preferably, BCS assessment must be performed by the same observer for maintaining evaluation consistency. The continual BCS estimation (i.e., estimations at different time points) allows close monitoring of the herd’s nutritional status (Houghton et al., 1990). Furthermore, a correct BCS ensures an optimal postpartum interval until the return of estrus and greater pregnancy rates (Selk et al., 1988; Renquist et al., 2006). Therefore, BCS estimation plays an essential role in the nutritional planning and management of the cow–calf operation systems. During the peripartal period, BCS is decreased because of the reduction in BW (Wang et al., 2019). In our study, the BCS scale from 1 to 9 was utilized, with 1 being an emaciated dam and 9 an over fat dam (Renquist et al., 2006). The reduction in BCS in Angus × Simmental dams from late gestation to early weaning in our study is a normal outcome due to the reduction in DMI during late gestation and the high nutritional demand during the lactation period. Primiparous dams require a superior-quality diet to fulfill metabolic requirements during lactation. During lactation period, nutrients from diet are directed toward recovering BW and lactation in multiparous dams; whereas primiparous dams’ nutritional requirements are directed toward recovering BW, lactation, and growing (Linden et al., 2014). Most studies investigating the effects of methionine on beef cattle used a methionine hydroxy analog; however, a growing area of research is currently utilizing RPM in their experiments partly due to the vast information available about its benefits on dairy cattle. The metabolism of methionine hydroxyl analog and protected DL-Met occurs differently in the animal’s body (FEEDAP, 2012). A recent study showed the lack of effect of RPM supplementation on gestating cows, in which authors reported similar BW of gestating Angus crossbreed cows consuming 9.5 g/hd/d of 2-hydroxy-4(methylthio)-butanoic acid (10 g/hd/d; 3.7 g/hd/d of metabolizable Met) compared to control cows. The crude protein (**CP**) in this experiment was 8.2% of the diet (Silva et al., 2021). Similarly, another experiment in which Angus × Simmental cows received a diet with 12.1% CP and methionine hydroxy analog did not show differences in BW compared with cows without supplementation (Clements et al., 2017). Therefore, we suggest that supplementation with Met in diets with adequate CP does not alter animal performance on gestating beef dams.

#### Offspring BW and ADG

After calving, primiparous dams’ nutrient partitioning is directed to growing and lactation. The greater energy requirement could be partly met by a greater DMI (as a percent of BW) compared with mature cows. Different nutritional and management strategies could be applied to ensure proper heifer growth, return to cyclicity, and lactation. For example, protein and energy supplementation and early weaning are widely utilized in grazing cow–calf operation systems. However, milk yield is significantly lower in primiparous dams compared with multiparous cows of the same breed (Fiss and Wilton, 1992). In our study, early weaning and creep feeding strategies were applied in order to improve calves’ growth. Creep feeding helps offspring’s performance not only during the lactation period but also provides a carry-over effect, positively affecting productive characteristics such as feedlot performance and carcass quality (e.g., marbling score; Myers et al., 1999). Interestingly and contrary to our expectations, the maternal supplementation with RPM did not impact offspring performance during lactation. In a previous experiment, primiparous beef dams receiving annual rye hay with ground corn, soybean meal, RPM, and protected lysine showed positive results regarding milk production. However, offspring born to dams supplemented with RPM did not present differences in animal’s performance (e.g., BW and ADG) compared to calves born to dams not receiving supplementation, suggesting that RPM supplementation did not improve milk production in primiparous dams (Hess et al., 1998).

After weaning, calves born to RPM dams received RPM until the end of the study. Our results indicate that RPM supplementation does not alter growth on Angus × Simmental calves. The offspring’s diet was formulated to meet NRC requirements ; therefore, methionine was not limiting in the diet and could not have impacted body composition patterns of growth which led to changes in BW (i.e., skeletal muscle production).

### Blood metabolites

Numerous metabolic, physiological, and endocrinal changes occur during weaning. Glucose is the main energy source for ruminants, and up to 50% of glucose is synthetized from propionate in ruminants. In addition, endogenous gluconeogenesis from glycerol could occur up to 40% in fasted animals (Oksbjerg et al., 2004). After weaning, circulating glucose concentration decreases significantly in ruminants due to the removal of liquid feed with high lactose content (e.g., maternal milk) from the diet, which presents a superlative nutritive value (Quigley et al., 1991). As expected, our study showed that early weaning reduced serum glucose concentration in all calves. From days 25 to 100, circulating glucose concentration remained stable at normal levels for beef cattle (~60 to 100 mg/dL) in all calves (Rumsey et al., 1999).

Albumin is the most abundant protein found in mammals’ blood and is responsible for regulating blood volume and protein transport. It is mainly synthesized in the liver, usually serving as a liver health biomarker (Osorio et al., 2014b). In our study, maternal supplementation with RPM did not affect albumin concentration at early weaning. Previous evidence reported a tendency for a slight reduction in albumin concentration for Holstein calves born to dams supplemented with RPM compared with those without RPM supplementation from birth to day 50 (Jacometo et al., 2016). Albumin concentration decreased between days 25 and 50; however, the supplementation with RPM did not affect albumin serum concentration. Likewise, Hill et al. (2008) evaluated the inclusion of different levels of methionine in young dairy calves. The authors reported no difference in albumin concentration among different methionine inclusion in the diet. Changes in albumin concentration could be associated with the change of the diet, from lactation with creep-feeding access to a roughage and early wean feed supplement, as reported by Lohakare et al. (2012). Most importantly, calves had circulating albumin within the normal range (2.7 to 3.9 g/dL) as reported by Keay and Doxey (1983) and Otter (2013), suggesting an adequate liver health status regardless of supplementation with RPM.

Furthermore, AST is the enzyme that catalyzes the transamination of aspartate and α-ketoglutarate into oxaloacetate and glutamate; whereas ALT is the enzyme responsible for converting alanine and α-ketoglutarate into pyruvate and glutamate. The serum activity of AST and ALT are also analyzed for identifying hepatocellular injury (Vuppalanchi and Chalasani, 2018). Remarkably, a previous study showed that maternal supplementation with RPM resulted in a lower concentration of offspring AST at birth in Holstein calves. However, after colostrum intake, AST concentration remained similar compared with control calves until 50 d of life (Jacometo et al., 2016). The concentration of ALT significantly increased from days 1 to 25, and from days 50 to 100. In accordance with our results, a previous study conducted by Suzuki et al. (2016) found greater ALT concentration in early-weaned Holstein calves compared with others of similar age that were lactating. These results indicate that AST and ALT concentration changes after weaning were not severe and were at normal levels as previous studies (Pavlík et al., 2009; Jacometo et al., 2016).

Finally, serum ALP was analyzed for identifying liver health damage. ALP is a plasma membrane-bound glycoprotein enzyme produced by the liver and bone, and its main function is to hydrolyze phosphate monoesters (Sharma et al., 2014). Calves born to dams receiving RPM showed a slightly greater concentration of circulating ALP. This difference may be explained by a possible greater bone growth in RPM calves compared with those in CTRL from birth to weaning (Yamaguchi and Yamaguchi, 1986; Chiba et al., 2020). Most importantly, calves did not exceed the maximum reference value of 1,200 U/L (20 µkat/L; Dresler et al., 2016). Consequently, based on the previous evidence and our results, we suggest that all calves were not exposed to severe stress during the experiment.

### Gene expression

#### Adipogenic genes

Currently, the price of beef in the United States is driven mostly by the intramuscular fat content or marbling. Therefore, several genetic and nutritional strategies have been applied in beef production systems during the last several decades for improving this trait in fattening animals. In addition to the vast impact of stocker, backgrounding, and finishing phase management and nutrition on marbling (Owens and Gardner, 2000; Lancaster et al., 2014), there is growing evidence in literature showing that maternal nutrition plays a key role in mature adipose tissue deposition (Du et al., 2013). More specifically, maternal nutrient restriction occurring during early to mid-gestation results in reduced skeletal muscle mass and increased fat content in the offspring (Zhu et al., 2006; Long et al., 2012). Conversely, intrauterine nutrient restriction during the last trimester of gestation reduces fetal adipose tissue content (Symonds et al., 2004). For example, a study conducted by Underwood et al. (2010) showed that maternal plane of nutrition during mid to late gestation affects carcass quality. In this study, Angus mature cows were exposed to native range pasture or improved pasture from 120 to 150 d through 180 to 210 d of gestation. Steers born to cows on improved pasture had greater final BW, hot carcass weight, 12th rib fat thickness, but there was no significant difference in marbling score. However, Larson et al. (2009) showed that steers born to Red Angus × Simmental mature cows receiving protein supplementation during late gestation had greater marbling content and quality grade, and a tendency to have higher empty body fat content compared with steers born to dams without protein supplementation.

With the use of current technology, adipose tissue formation can be explained and predicted by the expression of genes that codify proteins involved in such process. *PPARg* is the essential and primordial transcription factor of nuclear hormone receptors for adipogenesis in mammals. Therefore, *PPARg* is considered the “master regulator” of adipogenesis and lipogenesis, and can control the gene expression of other adipogenic factors (Wu et al., 1999). It has been demonstrated that *PPARg* plays an essential role in intramuscular fat accumulation, and its level of expression is associated with nutritional status and breed. For example, Moisá et al. (2014) reported the crucial role of *PPARg* in the early stages of cattle growth and final carcass quality. In this study, authors showed a greater number of carcasses with “high choice” quality (according to U.S. beef quality standard) on early-weaned steers compared with conventional weaned steers. Interestingly, the same study showed a greater expression of *PPARg* and *CEBPA*, which are key regulators of adipogenesis, on early-weaned calves, suggesting their important role in the process of intramuscular fat deposition that led to a greater percentage of “high choice” carcass quality grade. Similarly, a previous study conducted by Duarte et al. (2013) compared the expression of adipogenic factors in Wagyu, a breed known for its high intramuscular fat content, vs. Angus steers. There was a greater expression of *PPARg* in Wagyu steers than Angus steers; nevertheless, authors reported that Wagyu steers had a greater abundance of mesenchymal progenitor cells from which adipocytes and fibroblasts are differentiated. Thus, adipogenic differentiation occurred to a greater extent in Wagyu compared with Angus cattle. Another factor associated with fatty acid composition is the LPL. More specifically, *LPL* regulates the lipolysis of triglycerides enhancing the accumulation of adipose tissue (Pethick et al., 2004; Oh et al., 2013).

Furthermore, CEBPs are transcription regulators involved in adipogenic and lipogenic processes. The CEBP family comprises at least five proteins: CEBPA, CEBPB, CEBPD, CEBPE, and CEBPG. Each of these factors is present in almost all cell types as transcription factors, and they are involved in specific biologic mechanisms (Wedel and Lömsziegler-Heitbrock, 1995). In our study, we analyzed the expression of *CEBPD* and *CEBPG* due to their role in adipose tissue development in growing animals. It has been previously shown that *CEBPD* acts as an upstream regulator of *PPAR*g in mammals (Cao et al., 2015; Małodobra-Mazur et al., 2020). Similarly, *CEBPG* is highly expressed during the early stages of adipocyte differentiation (Bachmeier and Löffler, 1997). In our study, there was an upregulation of *PPARg*, *CEBPD*, and *LPL* at the end of the study in calves born to dams receiving RPM compared with CTRL group. Interestingly, the expression of *CEBPD* could be associated with a lower oxidative stress condition (Banerjee et al., 2016). In our study, we detected a greater *CEBPD* expression at day 100, and this upregulation could be associated with the greater expression of *SOD2* and *NOS3*, which are oxidative stress-related genes involved in the protection of the cell against radical ions (Jackson et al., 2007). Maternal parity has been identified as a major contributor to fat accumulation, being the progeny of primiparous dams who have greater adipose tissue content compared to those born to multiparous dams (Symonds et al., 2004). It was previously known that brown adipose tissue ablation causes an increment in total body lipid, increasing the probability of obesity (Lowell et al., 1993). A posterior study confirming this statement on ruminants reported that primiparous ewes’ offspring present more fat mass than those born to multiparous ewes due to the loss of brown adipose tissue (Hyatt et al., 2010). Therefore, calves born to primiparous dams may be more prone to experience upregulation in adipogenic-related genes (i.e., *PPARg*, *LPL*, and *CEBPD*) than those born to multiparous dams. In addition, reports indicate that *PPARg* expression can be altered by the diet received. For example, supplementation with methionine increases PPARg protein abundance in adipose tissue of Holstein cows 30 d after parturition (Liang et al., 2019). Similarly, a study conducted by Osorio et al. (2016) identified the upregulation of hepatic *PPARa*, another member of the peroxisome proliferator-activated receptor family, as a result of greater methylation due to the supplementation of RPM (Smartamine) on peripartal Holstein cows. Even though there may exist differences in adipogenic genes among tissues, Moisá et al. (2014) showed that dietary changes on beef steers can impact adipogenic gene expression in skeletal muscle of beef cattle. Calves born to primiparous dams could be more susceptible to experience methylation in their genome, including adipogenesis-related genes, as shown by the upregulation in *DNMT1* among time points. Since calves in our study were exposed to methionine through maternal feeding (e.g., uterus and milk) and directly after weaning, supplementation of RPM might have a direct impact on adipogenic genes on RPM group, upregulating the target genes analyzed in our study.

#### Oxidative stress and DNA methylation

Oxidative stress in a detrimental condition caused by an imbalance in the redox status of the cell. The compounds causing oxidative stress are superoxide (O_2_^−^) and nitric oxide (NO^−^) that disrupt redox signaling and molecular damage (Sies, 2015). Reactive oxygen species (**ROS**) results from the reduction of oxygen into reduced oxygen (**O**_**2**_^**−**^), hydrogen peroxide (**H**_**2**_**O**_**2**_), and hydroxyl radicals (**OH**^**−**^). SOD family plays a key role in scavenging free radicals in the cell. Mammals present three isoforms of SOD and they differ in their composition and site of action: SOD1 is located mainly in the cytosol and the intermembrane space of mitochondria to a lesser extent and is formed mainly by Cu/Zn, SOD2 is formed by Mn and is located in the mitochondrial matrix, and SOD3 is located at the extracellular matrix (Fukai and Ushio-Fukai, 2011). The main function of SODs is to cause spontaneous dismutation of O^−^ resulting in the formation of H_2_O_2._ Furthermore, SODs inhibit the formation of ONOO^−^. Since the major site of formation of O_2_^−^ takes place in the mitochondrial respiratory chain, measuring the expression of *SOD2* serves as a reliable indicator of ROS regulation in the cell (Hu et al., 2005). In addition, compounds involved in the trans-sulfuration pathway are also related to oxidative stress regulation, such as homocysteine, which is a product of methionine. One of the effects of homocysteine is to inhibit the production and release of nitric oxide (Li et al., 2007). Nitric oxide is synthesized by nitric oxide synthases isoforms, such as nitric oxide synthase 1 (**NOS1**), NOS2, and NOS3. First, the enzyme highly present in neural tissue is NOS1, which is also known as “nNOS”. Second, NOS2 is abundant in macrophages, and it is known as inducible NOS. Finally, NOS3 is involved in muscular and vascular tone. Since NOS3 is an endothelial nitric oxide isoform that also modulates muscular oxygen consumption and microvascular blood flow, this enzyme is usually referred as “eNOS” (Kobzik et al., 1995; Harrison, 1997; Mattila and Thomas, 2014).

Interestingly, in accordance with a previous study from our lab (Alfaro et al., 2020), after RPM supplementation on heifers for a period of 45 d, *SOD2* was downregulated. In this study, calves receiving RPM had a downregulation of *SOD2* from days 25 to 50 suggesting a greater capability of the cell to cope against redox imbalance. Similarly, Osorio et al. (2014a) showed a downregulation of hepatic *SOD1* but a stable expression of *SOD2* in peripartal Holstein cows receiving supplemented Smartamine. The authors suggested that methionine could be involved in the dismutation mechanism of the cell, lowering the oxidative stress status. However, contrary to previous results, our study indicates that the expression of *SOD2* and *NOS3* in skeletal muscle were upregulated between days 50 and 100 in RPM and had a greater expression at day 100 compared with CTRL. Since calves were growing during the experiment, our results may suggest a possible connection between ROS action on skeletal muscle hypertrophy. Accordingly, a previous report showed that nitric oxide regulates the induction of hypertrophy in skeletal muscle by activating transient receptor potential cation channel, subfamily V, member 1 (***TRPV1***) in mice. The authors found that NO and ONOO^−^ indirectly activate the mammalian target of rapamycin, which is a kinase protein and the major factor for protein synthesis (Ito et al., 2013).

Supplementing one-carbon metabolism substrates enhance methylation processes in mammalian DNA. Methionine is an essential amino acid with critical importance in methylation processes because it is a precursor of *S*-adenosyl methionine, widely recognized as the universal methyl donor (Niculescu and Zeisel, 2002). Maternal diet can promote epigenetic modifications in the fetus and posterior offspring life. Epigenetic alterations can be enhanced by DNA or histone methylation. Therefore, increased dietary methionine directly affects methylation status in different tissues, including skeletal muscle (Chmurzynska, 2010). Recently, Liu et al. (2020) conducted a study where Brangus × Angus crossbred beef cows were supplemented with methionine from days −30 to +90 relative to the beginning of the breeding season. This study revealed that maternal supplementation of methionine during the peripartal period alters overall DNA methylation levels in offspring 30 d after birth. The enzymes capable of methylating DNA belong to the DNA-methyltransferase (**DNMT**) family. More specifically, DNMT1 plays an essential role in DNA methylation maintenance on daughter DNA strands and it is highly active during myogenesis, whereas DNTM3 enzymes are involved in de novo methylation (Liu et al., 2016). Even though the process of myogenesis and consequently the total skeletal muscle fiber number of all the calves in our study was expected to be completed at the moment of sampling, the supplementation with RPM upregulated the expression of *DNMT1* on RPM calves from days 50 to 100. A previous experiment conducted in humans identified changes in the methylation status of the epigenome due to changes in the diet. During this study, young men who were exposed to a high-fat overfeeding diet for 5 d had an upregulation in *DNMT1* and *DNMT3a* from skeletal muscle, suggesting that expression of *DNMT1* could be altered in growing and mature mammals by the diet consumed (Jacobsen et al., 2012). In addition, a recent experiment conducted by Iio et al. (2021) found a novel function of *DNMT1* during muscle regeneration in mature male mice. These results suggest that *DNMT1* may be involved in other methylation processes in addition to cell division. Further research is needed to elucidate the mechanisms behind the upregulation of *DNMT1* in calves born to primiparous dams, and if this greater expression has an impact on skeletal muscle hypertrophy.

## Conclusion

Herd management is essential in cow–calf operations, and more critically, during gestation. Management practices, including diet formulation, have a substantial impact on gestating dam and offspring’s postnatal growth and development. Our study showed similar performance parameters (e.g., BW and BCS) on calves supplemented with RPM during the last trimester of gestation and the first ~80 d of lactation compared with those receiving a CTRL diet. Similarly, growth parameters did not differ in calves born to primiparous dams receiving RPM compared with CTRL during lactation and after weaning. Blood metabolites that serve as biomarkers for liver health status were in the normal range levels in all treatment groups. Remarkably, calves born to RPM dams had an upregulation in *PPARg*, *CEBPD*, and *LPL* at the end of the study compared with those in CTRL. These results suggest that calves that had greater exposure to methionine in utero, milk, and direct supplementation after weaning with RPM could potentially result in greater adipose tissue development and increasing marbling during the finishing period. However, caution must be exercised when interpreting our data since it was not possible to confirm whether the upregulation of these genes was further translated into better carcass quality. Finally, RPM calves had greater expression of *SOD2*, *NOS3,* and *DNMT1* at day 100 compared with CTRL. Our results are in accordance with previous evidence that show alteration in DNA methylation due to the inclusion of supplemental RPM. Furthermore, the greater expression of oxidative stress-related genes could be an indicator of a greater hypertrophy process in skeletal muscle.

## Supplementary Material

skae006_suppl_Supplementary_Tables_S1-S4

## Abbreviations

ADG: average daily gain;
ALP: alkaline phosphatase;
ALT: alanine transaminase;
AST: aspartate transaminase
BCS: body condition score
BW: body weight
cDNA: complementary DNA
CEBPD: CCAAT/enhancer binding protein delta
CEBPG: CCAAT/enhancer binding protein gamma
CP: crude protein
DMI: dry matter intake
DNMT1: DNA methyltransferase 1
LPL: lipoprotein lipase
MTG1: mitochondrial ribosome-associated GTPase 1
PPARg: peroxisome proliferator-activated receptor gamma
qPCR: quantitative polymerase chain reaction
ROS: reactive oxygen species
RPM: rumen-protected methionine
RPS15A: ribosomal protein S15a
SOD2: superoxide dismutase 2
UXT: ubiquitously expressed prefoldin like chaperone.

## References

[CIT0001] Alfaro, G. F., T. E. Novak, S. P. Rodning, and S. J. Moisá. 2020. Preconditioning beef cattle for long-duration transportation stress with rumen-protected methionine supplementation: a nutrigenetics study. PLoS One. 15:e0235481. doi:10.1371/journal.pone.0235481 32614880 PMC7332072

[CIT0002] Bachmeier, M., and G. Löffler. 1997. The effect of platelet-derived growth factor and adipogenic hormones on the expression of CCAAT/Enhancer-binding proteins in 3T3-L1 cells in serum-free conditions. Eur. J. Biochem. 243:128–133. doi:10.1111/j.1432-1033.1997.0128a.x 9030731

[CIT0003] Banerjee, S., N. Aykin-Burns, K. J. Krager, S. K. Shah, S. B. Melnyk, M. Hauer-Jensen, and S. A. Pawar. 2016. Loss of C/EBPδ enhances IR-induced cell death by promoting oxidative stress and mitochondrial dysfunction. Free Radic. Biol. Med. 99:296–307. doi:10.1016/j.freeradbiomed.2016.08.022 27554969 PMC5673253

[CIT0004] Bowling, J. L., and A. Katayev. 2010. An evaluation of the Roche Cobas c 111. Lab. Med. 41:398–402. doi:10.1309/lm6t8d1lkqxvncac

[CIT0005] Cantoni, G. L. 1975. Biological methylation: selected aspects. Annu. Rev. Biochem. 44:435–451. doi:10.1146/annurev.bi.44.070175.002251 1094914

[CIT0006] Cao, Y., S. A. Gomes, E. B. Rangel, E. C. Paulino, T. L. Fonseca, J. Li, M. B. Teixeira, C. H. Gouveia, A. C. Bianco, M. S. Kapiloff, et al. 2015. S-Nitrosoglutathione reductase–dependent PPARγ denitrosylation participates in MSC-derived adipogenesis and osteogenesis. J. Clin. Invest. 125:1679–1691. doi:10.1172/JCI73780 25798618 PMC4396480

[CIT0007] Chiba, A., K. Hatate, R. Onomi, C. Kawashima, M. Hanada, T. Moriyama, A. Goto, and N. Yamagishi. 2020. Consecutive changes in serum alkaline phosphatase isoenzyme 3 activities in Holstein heifers during the first 18 months of life. J. Vet. Med. Sci. 82:1643–1647. doi:10.1292/jvms.20-0306 32963214 PMC7719893

[CIT0008] Chmurzynska, A. 2010. Fetal programming: link between early nutrition, DNA methylation, and complex diseases. Nutr. Rev. 68:87–98. doi:10.1111/j.1753-4887.2009.00265.x 20137054

[CIT0009] Clements, A. R., F. A. Ireland, T. Freitas, H. Tucker, and D. W. Shike. 2017. Effects of supplementing methionine hydroxy analog on beef cow performance, milk production, reproduction, and preweaning calf performance. J. Anim. Sci. 95:5597–5605. doi:10.2527/jas2017.1828 29293801 PMC6292334

[CIT0010] Dresler, S., J. Illek, and L. Zeman. 2016. Effects of organic zinc supplementation in weaned calves. Acta Vet. Brno 85:49–54. doi:10.2754/avb201685010049

[CIT0011] Drouillard, J. S. 2018. Current situation and future trends for beef production in the United States of America — a review. Asian-Australas. J. Anim. Sci. 31:1007–1016. doi:10.5713/ajas.18.0428 29973030 PMC6039332

[CIT0012] Du, M., Y. Huang, A. K. Das, Q. Yang, M. S. Duarte, M. V. Dodson, and M. -J. Zhu. 2013. Meat science and muscle biology symposium: manipulating mesenchymal progenitor cell differentiation to optimize performance and carcass value of beef cattle. J. Anim. Sci. 91:1419–1427. doi:10.2527/jas.2012-5670 23100595

[CIT0013] Du, M., B. Wang, X. Fu, Q. Yang, and M. -J. Zhu. 2015. Fetal programming in meat production. Meat Sci. 109:40–47. doi:10.1016/j.meatsci.2015.04.010 25953215

[CIT0014] Duarte, M. S., P. V. R. Paulino, A. K. Das, S. Wei, N. V. L. Serão, X. Fu, S. M. Harris, M. V. Dodson, and M. Du. 2013. Enhancement of adipogenesis and fibrogenesis in skeletal muscle of Wagyu compared with Angus cattle. J. Anim. Sci. 91:2938–2946. doi:10.2527/jas.2012-5892 23508025

[CIT0015] Feedap, F. 2012. Scientific opinion on DL-methionine, DL-methionine sodium salt, the hydroxy analogue of methionine and the calcium salt of methionine hydroxy analogue in all animal species; on the isopropyl ester of methionine hydroxy analogue and DL-methionine technically pure protected with copolymer vinylpyridine/styrene in dairy cows; and on DL-methionine technically pure protected with ethylcellulose in ruminants. EFSA J. 10:2623. doi:10.2903/j.efsa.2012.2623 PMC13151806 42112027

[CIT0016] Fiss, C. F., and J. W. Wilton. 1992. Contribution of breed, cow weight, and milk yield to the traits of heifers and cows in four beef breeding systems. J. Anim. Sci. 70:3686–3696. doi:10.2527/1992.70123686x 1474008

[CIT0017] Fukai, T., and M. Ushio-Fukai. 2011. Superoxide dismutases: role in redox signaling, vascular function, and diseases. Antioxid. Redox Signal. 15:1583–1606. doi:10.1089/ars.2011.3999 21473702 PMC3151424

[CIT0018] Harrison, D. G. 1997. Cellular and molecular mechanisms of endothelial cell dysfunction. J. Clin. Invest. 100:2153–2157. doi:10.1172/JCI119751 9410891 PMC508409

[CIT0019] Hess, B. W., E. J. Scholljegerdes, S. A. Coleman, and J. E. Williams. 1998. Supplemental protein plus ruminally protected methionine and lysine for primiparous beef cattle consuming annual rye hay. J. Anim. Sci. 76:1767–1777. doi:10.2527/1998.7671767x 9690631

[CIT0020] Hill, T. M., H. G. Bateman, J. M. Aldrich, R. L. Schlotterbeck, and K. G. Tanan. 2008. Optimal concentrations of lysine, methionine, and threonine in milk replacers for calves less than five weeks of age. J. Dairy Sci. 91:2433–2442. doi:10.3168/jds.2007-0610 18487666

[CIT0021] Houghton, P. L., R. P. Lemenager, G. E. Moss, and K. S. Hendrix. 1990. Prediction of postpartum beef cow body composition using weight to height ratio and visual body condition score. J. Anim. Sci. 68:1428–1437. doi:10.2527/1990.6851428x

[CIT0022] Hu, Y., D. G. Rosen, Y. Zhou, L. Feng, G. Yang, J. Liu, and P. Huang. 2005. Mitochondrial manganese-superoxide dismutase expression in ovarian cancer: role in cell proliferation and response to oxidative stress. J. Biol. Chem. 280:39485–39492. doi:10.1074/jbc.M503296200 16179351

[CIT0023] Hyatt, M. A., D. H. Keisler, H. Budge, and M. E. Symonds. 2010. Maternal parity and its effect on adipose tissue deposition and endocrine sensitivity in the postnatal sheep. J. Endocrinol. 204:173–179. doi:10.1677/JOE-09-0358 19934248 PMC2807923

[CIT0024] Iio, H., T. Kikugawa, Y. Sawada, H. Sakai, S. Yoshida, Y. Yanagihara, A. Ikedo, N. Saeki, S. Fukada, T. Saika, et al. 2021. DNA maintenance methylation enzyme Dnmt1 in satellite cells is essential for muscle regeneration. Biochem. Biophys. Res. Commun. 534:79–85. doi:10.1016/j.bbrc.2020.11.116 33310192

[CIT0025] Ito, N., U. T. Ruegg, A. Kudo, Y. Miyagoe-Suzuki, and S. Takeda. 2013. Activation of calcium signaling through Trpv1 by nNOS and peroxynitrite as a key trigger of skeletal muscle hypertrophy. Nat. Med. 19:101–106. doi:10.1038/nm.3019 23202294

[CIT0026] Jackson, M. J., D. Pye, and J. Palomero. 2007. The production of reactive oxygen and nitrogen species by skeletal muscle. J. Appl. Physiol. (1985). 102:1664–1670. doi:10.1152/japplphysiol.01102.2006 17082364

[CIT0027] Jacobsen, S. C., C. Brøns, J. Bork-Jensen, R. Ribel-Madsen, B. Yang, E. Lara, E. Hall, V. Calvanese, E. Nilsson, S. W. Jørgensen, et al. 2012. Effects of short-term high-fat overfeeding on genome-wide DNA methylation in the skeletal muscle of healthy young men. Diabetologia. 55:3341–3349. doi:10.1007/s00125-012-2717-8 22961225

[CIT0028] Jacometo, C. B., Z. Zhou, D. Luchini, E. Trevisi, M. N. Corrêa, and J. J. Loor. 2016. Maternal rumen-protected methionine supplementation and its effect on blood and liver biomarkers of energy metabolism, inflammation, and oxidative stress in neonatal Holstein calves. J. Dairy Sci. 99:6753–6763. doi:10.3168/jds.2016-11018 27209133

[CIT0029] Keay, G., and D. L. Doxey. 1983. Serum albumin values from healthy cattle, sheep and horses determined by the immediate bromocresol green reaction and by agarose gel electrophoresis. Res. Vet. Sci. 35:58–60. doi:10.1016/s0034-5288(18)32203-3 6622845

[CIT0030] Kobzik, L., B. Stringer, J. L. Balligand, M. B. Reid, and J. S. Stamler. 1995. Endothelial type nitric oxide synthase in skeletal muscle fibers: mitochondrial relationships. Biochem. Biophys. Res. Commun. 211:375–381. doi:10.1006/bbrc.1995.1824 7540837

[CIT0031] Kowalski, Z. M., H. N. Pisulewski, and M. Gorgulu. 2003. Effects of protected methionine and variable energy supply on lactational responses in dairy cows fed grass silage-based diets. J. Anim. Feed Sci. 12:451-464. doi:10.22358/jafs/67722/2003.

[CIT0032] Lancaster, P. A., C. R. Krehbiel, and G. W. Horn. 2014. A meta-analysis of effects of nutrition and management during the stocker and backgrounding phase on subsequent finishing performance and carcass characteristics. Prof. Anim. Sci. 30:602–612. doi:10.15232/pas.2014-01330

[CIT0033] Larson, D. M., J. L. Martin, D. C. Adams, and R. N. Funston. 2009. Winter grazing system and supplementation during late gestation influence performance of beef cows and steer progeny. J. Anim. Sci. 87:1147–1155. doi:10.2527/jas.2008-1323 18997078 PMC7110207

[CIT0034] Li, P., Y. -L. Yin, D. Li, S. W. Kim, and G. Wu. 2007. Amino acids and immune function. Br. J. Nutr. 98:237–252. doi:10.1017/S000711450769936X 17403271

[CIT0035] Liang, Y., F. Batistel, C. Parys, and J. J. Loor. 2019. Methionine supply during the periparturient period enhances insulin signaling, amino acid transporters, and mechanistic target of rapamycin pathway proteins in adipose tissue of Holstein cows. J. Dairy Sci. 102:4403–4414. doi:10.3168/jds.2018-15738 30879817

[CIT0036] Linden, D. R., E. C. Titgemeyer, K. C. Olson, and D. E. Anderson. 2014. Effects of gestation and lactation on forage intake, digestion, and passage rates of primiparous beef heifers and multiparous beef cows1. J. Anim. Sci. 92:2141–2151. doi:10.2527/jas.2013-6813 24663177

[CIT0037] Liu, R., K. -Y. Kim, Y. -W. Jung, and I. -H. Park. 2016. Dnmt1 regulates the myogenic lineage specification of muscle stem cells. Sci. Rep. 6:35355. doi:10.1038/srep35355 27752090 PMC5082760

[CIT0038] Liu, L., R. Amorín, P. Moriel, N. DiLorenzo, P. A. Lancaster, and F. Peñagaricano. 2020. Differential network analysis of bovine muscle reveals changes in gene coexpression patterns in response to changes in maternal nutrition. BMC Genomics. 21:684. doi:10.1186/s12864-020-07068-x 33008289 PMC7531131

[CIT0039] Lohakare, J. D., H. van de Sand, K. Gerlach, A. Hosseini, M. Mielenz, H. Sauerwein, M. Pries, and K. -H. Südekum. 2012. Effects of limited concentrate feeding on growth and blood and serum variables, and on nutrient digestibility and gene expression of hepatic gluconeogenic enzymes in dairy calves. J. Anim. Physiol. Anim. Nutr. 96:25–36. doi:10.1111/j.1439-0396.2010.01117.x 21210862

[CIT0040] Long, N. M., C. B. Tousley, K. R. Underwood, S. I. Paisley, W. J. Means, B. W. Hess, M. Du, and S. P. Ford. 2012. Effects of early- to mid-gestational undernutrition with or without protein supplementation on offspring growth, carcass characteristics, and adipocyte size in beef cattle. J. Anim. Sci. 90:197–206. doi:10.2527/jas.2011-4237 21908644

[CIT0041] Lowell, B. B., V. S-Susulic, A. Hamann, J. A. Lawitts, J. Himms-Hagen, B. B. Boyer, L. P. Kozak, and J. S. Flier. 1993. Development of obesity in transgenic mice after genetic ablation of brown adipose tissue. Nature. 366:740–742. doi:10.1038/366740a0 8264795

[CIT0042] Małodobra-Mazur, M., A. Cierzniak, D. Pawełka, K. Kaliszewski, J. Rudnicki, and T. Dobosz. 2020. Metabolic differences between subcutaneous and visceral adipocytes differentiated with an excess of saturated and monounsaturated fatty acids. Genes. 11:1092. doi:10.3390/genes11091092 32962087 PMC7563871

[CIT0043] Mattila, J. T., and A. C. Thomas. 2014. Nitric oxide synthase: non-canonical expression patterns. Front. Immunol. 5:478. doi:10.3389/fimmu.2014.00478 25346730 PMC4191211

[CIT0044] Misciatteilli, L., V. F. Kristensen, M. Vestergaard, M. R. Welsbjerg, K. Sejrsen, and T. Hvelplund. 2003. Milk production, nutrient utilization, and endocrine responses to increased postruminal lysine and methionine supply in dairy cows. J. Dairy Sci. 86:275–286. doi:10.3168/jds.s0022-0302(03)73606-6 12613871

[CIT0045] Moisá, S. J., D. W. Shike, D. B. Faulkner, W. T. Meteer, D. Keisler, and J. J. Loor. 2014. Central role of the PPARγ gene network in coordinating beef cattle intramuscular adipogenesis in response to weaning age and nutrition. Gene Regul. Syst. Biol. 8:17–32. doi:10.4137/GRSB.S11782 PMC3894150 24516329

[CIT0046] Myers, S. E., D. B. Faulkner, F. A. Ireland, L. L. Berger, and D. F. Parrett. 1999. Production systems comparing early weaning to normal weaning with or without creep feeding for beef steers. J. Anim. Sci. 77:300–310. doi:10.2527/1999.772300x 10100657

[CIT0047] Niculescu, M. D., and S. H. Zeisel. 2002. Diet, methyl donors and DNA methylation: interactions between dietary folate, methionine and choline. J. Nutr. 132:2333S–2335S. doi:10.1093/jn/132.8.2333S 12163687

[CIT0048] Oh, D., B. La, Y. Lee, Y. Byun, J. Lee, G. Yeo, and J. Yeo. 2013. Identification of novel single nucleotide polymorphisms (SNPs) of the lipoprotein lipase (LPL) gene associated with fatty acid composition in Korean cattle. Mol. Biol. Rep. 40:3155–3163. doi:10.1007/s11033-012-2389-y 23271120

[CIT0049] Oksbjerg, N., F. Gondret, and M. Vestergaard. 2004. Basic principles of muscle development and growth in meat-producing mammals as affected by the insulin-like growth factor (IGF) system. Domest. Anim. Endocrinol. 27:219–240. doi:10.1016/j.domaniend.2004.06.007 15451071

[CIT0050] Osorio, J. S., P. Ji, J. K. Drackley, D. Luchini, and J. J. Loor. 2014a. Smartamine M and MetaSmart supplementation during the peripartal period alter hepatic expression of gene networks in 1-carbon metabolism, inflammation, oxidative stress, and the growth hormone–insulin-like growth factor 1 axis pathways. J. Dairy Sci. 97:7451–7464. doi:10.3168/jds.2014-8680 25282416

[CIT0051] Osorio, J. S., E. Trevisi, P. Ji, J. K. Drackley, D. Luchini, G. Bertoni, and J. J. Loor. 2014b. Biomarkers of inflammation, metabolism, and oxidative stress in blood, liver, and milk reveal a better immunometabolic status in peripartal cows supplemented with Smartamine M or MetaSmart. J. Dairy Sci. 97:7437–7450. doi:10.3168/jds.2013-7679 25282419

[CIT0052] Osorio, J. S., C. B. Jacometo, Z. Zhou, D. Luchini, F. C. Cardoso, and J. J. Loor. 2016. Hepatic global DNA and peroxisome proliferator-activated receptor alpha promoter methylation are altered in peripartal dairy cows fed rumen-protected methionine. J. Dairy Sci. 99:234–244. doi:10.3168/jds.2015-10157 26585478

[CIT0053] Otter, A. 2013. Diagnostic blood biochemistry and haematology in cattle. In Practice. 35:7–16. doi:10.1136/inp.e8719

[CIT0054] Owens, F., and B. Gardner. 2000. A review of the impact of feedlot management and nutrition on carcass measurements. J. Anim. Sci. 77:1–18. doi:10.2527/jas2000.00218812007700ES0034x.

[CIT0055] Pavlík, A., R. Zahrádková, D. Bureš, P. Jelínek, and Z. Havlíček. 2009. Indicators of the internal environment of gasconne calves during rearing. Acta Vet. Brno. 78:37–45. doi:10.2754/avb200978010037

[CIT0056] Pethick, D. W., G. S. Harper, and V. H. Oddy. 2004. Growth, development and nutritional manipulation of marbling in cattle: a review. Aust. J. Exp. Agric. 44:705–715. doi:10.1071/ea02165

[CIT0057] Quigley, J. D., L. A. Caldwell, G. D. Sinks, and R. N. Heitmann. 1991. Changes in blood glucose, nonesterified fatty acids, and ketones in response to weaning and feed intake in young calves. J. Dairy Sci. 74:250–257. doi:10.3168/jds.S0022-0302(91)78167-8 2030170

[CIT0059] Redifer, C. A., N. B. Duncan, and A. M. Meyer. 2021. Factors affecting placental size in beef cattle: Maternal and fetal influences. Theriogenology. 174:149–159. doi:10.1016/j.theriogenology.2021.08.015 34454320

[CIT0060] Renquist, B. J., J. W. Oltjen, R. D. Sainz, and C. C. Calvert. 2006. Relationship between body condition score and production of multiparous beef cows. Livest. Sci. 104:147–155. doi:10.1016/j.livsci.2006.04.004 16775073

[CIT0061] Rulquin, H., and L. Delaby. 1997. Effects of the energy balance of dairy cows on lactational responses to rumen-protected methionine. J. Dairy Sci. 80:2513–2522. doi:10.3168/jds.S0022-0302(97)76204-0 9361223

[CIT0062] Rumsey, T. S., S. Kahl, and T. H. Elsasser. 1999. Field method for monitoring blood glucose in beef cattle. J. Anim. Sci. 77:2194–2200. doi:10.2527/1999.7782194x 10461999

[CIT0063] Schwab, C. G. 2007. Protected proteins and amino acids for ruminants. In: Biotechnology in animal feeds and animal feeding. Weinheim, Germany: Wiley-VCH Verlag GmbH; p. 115–141. doi:10.1002/9783527615353.ch7

[CIT0064] Selk, G. E., R. P. Wettemann, K. S. Lusby, J. W. Oltjen, S. L. Mobley, R. J. Rasby, and J. C. Garmendia. 1988. Relationship among weight change, body condition and reproductive performance of range beef cows. J. Anim. Sci. 66:3153–3159. doi:10.2527/jas1988.66123153x 3230075

[CIT0065] Sharma, U., D. Pal, and R. Prasad. 2014. Alkaline phosphatase: an overview. Indian J. Clin. Biochem. 29:269–278. doi:10.1007/s12291-013-0408-y 24966474 PMC4062654

[CIT0066] Sies, H. 2015. Oxidative stress: a concept in redox biology and medicine. Redox Biol. 4:180–183. doi:10.1016/j.redox.2015.01.002 25588755 PMC4309861

[CIT0067] Silva, G. M., C. D. Chalk, J. Ranches, T. M. Schulmeister, D. D. Henry, N. DiLorenzo, J. D. Arthington, P. Moriel, and P. A. Lancaster. 2021. Effect of rumen-protected methionine supplementation to beef cows during the periconception period on performance of cows, calves, and subsequent offspring. Animal. 15:100055. doi:10.1016/j.animal.2020.100055 33516019

[CIT0068] Stanley, T. A., R. C. Cochran, E. S. Vanzant, D. L. Harmon, and L. R. Corah. 1993. Periparturient changes in intake, ruminal capacity, and digestive characteristics in beef cows consuming alfalfa hay2. J. Anim. Sci. 71:788–795. doi:10.2527/1993.713788x 8385090

[CIT0069] Suzuki, Y., S. Haga, M. Nakano, H. Ishizaki, M. Nakano, S. Song, K. Katoh, and S. Roh. 2016. Postweaning changes in the expression of chemerin and its receptors in calves are associated with the modification of glucose metabolism. J. Anim. Sci. 94:4600–4610. doi:10.2527/jas.2016-0677 27898966

[CIT0070] Symonds, M. E., S. Pearce, J. Bispham, D. S. Gardner, and T. Stephenson. 2004. Timing of nutrient restriction and programming of fetal adipose tissue development. Proc. Nutr. Soc. 63:397–403. doi:10.1079/pns2004366 15373949

[CIT0071] Underwood, K. R., J. F. Tong, P. L. Price, A. J. Roberts, E. E. Grings, B. W. Hess, W. J. Means, and M. Du. 2010. Nutrition during mid to late gestation affects growth, adipose tissue deposition, and tenderness in cross-bred beef steers. Meat Sci. 86:588–593. doi:10.1016/j.meatsci.2010.04.008 20659786

[CIT0072] Vuppalanchi, R., and N. Chalasani. 2018. 3 - Laboratory tests in liver disease. In: R. Saxena, editor. Practical Hepatic Pathology: A Diagnostic Approach. 2nd ed. Elsevier, Philadelphia. p. 43–53. doi:10.1016/B978-0-323-42873-6.00003-2

[CIT0073] Wang, Y., P. Huo, Y. Sun, and Y. Zhang. 2019. Effects of body condition score changes during peripartum on the postpartum health and production performance of primiparous dairy cows. Animals (Basel). 9:1159. doi:10.3390/ani9121159 31861177 PMC6940961

[CIT0074] Wedel, A., and H. W. Lömsziegler-Heitbrock. 1995. The C/EBP family of transcription factors. Immunobiology. 193:171–185. doi:10.1016/s0171-2985(11)80541-3 8530141

[CIT0075] Wu, Z., E. D. Rosen, R. Brun, S. Hauser, G. Adelmant, A. E. Troy, C. McKeon, G. J. Darlington, and B. M. Spiegelman. 1999. Cross-regulation of C/EBPα and PPARγ controls the transcriptional pathway of adipogenesis and insulin sensitivity. Mol. Cell 3:151–158. doi:10.1016/s1097-2765(00)80306-8 10078198

[CIT0076] Yamaguchi, M., and R. Yamaguchi. 1986. Action of zinc on bone metabolism in rats: increases in alkaline phosphatase activity and DNA content. Biochem. Pharmacol. 35:773–777. doi:10.1016/0006-2952(86)90245-5 3954786

[CIT0077] Zhu, M. J., S. P. Ford, W. J. Means, B. W. Hess, P. W. Nathanielsz, and M. Du. 2006. Maternal nutrient restriction affects properties of skeletal muscle in offspring. J. Physiol. 575:241–250. doi:10.1113/jphysiol.2006.112110 16763001 PMC1819430

