# Supplementary material for: Fetal programming effect of rumen-protected methionine on primiparous Angus × Simmental offspring’s performance and skeletal muscle gene expression
Source: J Anim Sci. 2024 Jan 10;102:skae006. doi: 10.1093/jas/skae006 (PMC10881097; doi:10.1093/jas/skae006)
Supplement: skae006_suppl_Supplementary_Tables_S1-S4 [file skae006_suppl_supplementary_tables_s1-s4.docx]

**Supplemental Table 1.** Gene ID, GenBank accession number, hybridization position, sequence and amplicon size of primers for *Bos Taurus* used to analyze gene expression by RT-qPCR.

| **Gene ID** | **Accession #** | **Gene** | **Primers^1^** | **Primers (5’-3’)** | **bp^2^** |  |
| --- | --- | --- | --- | --- | --- | --- |
| *Internal Controls* | |  |  |  |  |  |
| 509768 | NM_001025327.2 | *MTG1* | F.258 | CAACAAAATGGACCTGGCAGAT | 115 |  |
|  |  | *MTG1* | R.372 | CTTGACATTTTCATCCTTCACACAGT |  |  |
| 337888 | NM_001037443.2 | *RPS15A* | F.230 | GCAGGCTAAATAAGTGTGGAGTGA | 90 |  |
|  |  | *RPS15A* | R.319 | GGGATGGGAGCAGGTTATTCT |  |  |
| 525680 | NM_001037471.2 | *UXT* | F. 337 | CTGGCAGAAGCTCTCAAGTTCA | 100 |  |
|  |  | *UXT* | R. 436 | GGATATGGGCCTTGATATTCATG |  |  |
| *Genes of interest* | |  |  |  |  |  |
| 281496 | NM_201527.2 | *SOD2* | F 290 | AGAAGGGTGATGTTACAGCTCAGATAG | 93 |  |
|  |  | *SOD2* | R 382 | GATTTGTCCAGAAGATGCTGTGAT |  |  |
| 281993 | NM_181024.2 | *PPARG* | F. 439 | ACCCGATGGTTGCAGATTATAAG | 140 |  |
|  |  | *PPARG* | R. 578 | GGAGTTGGAAGGCTCTTCATGA |  |  |
| 280843 | NM_001075120.1 | *LPL* | F. 768 | CAAGTCGCCTTTCTCCTGATG | 105 |  |
|  |  | *LPL* | R. 872 | CATGCCCTACTGGTTTCTGGAT |  |  |
| 281678 | NM_001075676.1 | *CEBPD* | F.1440 | AGGAGATGGAAAGGACAGTCACA | 100 |  |
|  |  | *CEBPD* | R.1539 | AACGACTTTATTTATTCGTCCAGGTT |  |  |
| 617530 | NM_001034315.1 | *CEBPG* | F. 405 | AGGAACAACATGGCTGTGAAAA | 95 |  |
|  |  | *CEBPG* | R. 499 | TCATTCTCTTCCTTGAGCTGATTG |  |  |
| 281119 | NM_182651.2 | *DNMT1* | F. 1323 | TCTGGTTCAGCAAAGCCGATATAT | 105 |  |
|  |  | *DNMT1* | R. 1427 | ATCAAAACCAGCAATCCACCAT |  |  |
| 287024 | NM_181037.3 | *NOS3* | F. 3944 | CTCCGGAAGTATCTTATCTTGAAACC | 135 |  |
|  |  | *NOS3* | R. 4078 | AACTGTAATTGACAGCACTGGCTTAG |  |  |

^1^ Primer direction (F – forward; R – reverse) and hybridization position on the sequence.
^2^ Amplicon size in base pair (bp).

**Supplemental Table 2.** Sequencing results of PCR products from primers of genes designed for this experiment.

| Internal controls | | | | | | | | | |
| --- | --- | --- | --- | --- | --- | --- | --- | --- | --- |
| MTG1 | CGACCGAAATACCACCCTTGGAAGAGAGGCATAAAACATGTTTGTTTTTCCAACCTGTGTGAAGGATGA | | | | | | | | |
|  | AAATGTCAAGA |  |  |  |  |  |  |  |  |
| RPS15A | GACTGATATGAGTGTCACTCATAGTATCCTAGGATAGTGGCAGAATAACCTGCTCCCAGTCCCAACTCCA | | | | | | | | |
|  | CACTATTATTACGGTACGTTACGCATG | | |  |  |  |  |  |  |
| UXT | GTCTAAACGTCCCCTCCGGGGGCTCAGCGACAACCTTCACCAAGGGACTTCCATTGAATATTCAAGGGCC  CATTATTCCAA | | | | | | | | |
| Genes of interest | | | | | | | | | |
| SOD2 | CCGTTGCCCGGTCGTGGTAGTCTACGGGGTGCCATATCAATCACAGCATCTTCGTGACAAATCATAGAA | | | | | | | |  |
| PPARG | CAGTGACTCAGAGTACAAGTGCATGCAAAGTGGAGCCGTGTATCCCCACCTTATTATTCGTGAAAAGAC | | | | | | | | |
|  | TCGAGTCATAGTACAGTAAGCCTCAT | | |  |  |  |  |  |  |
| LPL | CGATGGATCAGTACCACATCACCAGGGGGTCACCAGGTCGAAGTATCGGAATCCAGGAAAACNCAGTA | | | | | | | | |
|  | GGCGCATGA |  |  |  |  |  |  |  |  |
| CEBPD | GACTTACTGCTACTGTCAGTACTTGTATATTTTCAAAGATTAACTGGACGAATAAATAAAGTCGTCCTAGCA | | | | | | | | |
| CEBPG | GCAGGTAGCAGCAGAGCGCAGGATACGCGTGCAGAGAGGTCAATCAGCTCAAGGGGAAGAGGAATGGAAA | | | | | | | | |
| DNMT1 | CGATACATCTCGCAGTGTATTATGGCAAAATTTTGGCATAAACGAATGGTGGATTGCTGGTTTCTGATAA | | | | | | | | |
| NOS3 | GTATCTATCATTCATATTTATTATTGAGATACCATAAGAGACTGGACCAGAAGTTAGGAGACCTATCTAAGA | | | | | | | | |

Best hits using BLASTN (http://ncbi.nlm.nih.gov) are shown.

**Supplemental Table 3.** Sequencing results of genes using BLASTN*

| **Gene transcript** | **Best hit in NCBI** |
| --- | --- |
| *CEBPD* | Bos taurus CCAAT enhancer binding protein delta (CEBPD), mRNA |
| *CEBPG* | Bos taurus CCAAT enhancer binding protein gamma (CEBPG), mRNA |
| *DNMT1* | Bos taurus DNA methyltransferase 1 (DNMT1), mRNA |
| *LPL* | [Bos taurus lipoprotein lipase (LPL), mRNA](https://blast.ncbi.nlm.nih.gov/Blast.cgi#alnHdr_115497163) |
| *MTG1* | Bos taurus mitochondrial ribosome associated GTPase 1 (MTG1), mRNA |
| *NOS3* | [Bos taurus nitric oxide synthase 3 (endothelial cell) (NOS3), mRNA](https://blast.ncbi.nlm.nih.gov/Blast.cgi#alnHdr_317008622) |
| *PPARG* | Bos taurus peroxisome proliferator activated receptor gamma (PPARG), mRNA |
| *RPS15A* | Bos taurus ribosomal protein S 15 a (RPS15A), mRNA |
| *SOD2* | Bos taurus superoxide dismutase 2 (SOD2), mRNA |
| *UXT* | Bos taurus ubiquitously expressed prefoldin like chaperone (UXT), mRNA |

* BLASTN from NCBI (http://ncbi.nlm.nih.gov)

**Supplemental Table 4.** Quantitative real time PCR performance among the 7 genes measured in skeletal muscle samples

| **Gene transcript** | **Median Ct^1^** | | **Median ∆Ct^2^** | **Slope^3^** | **(R^2^)^4^** | **Efficiency (%) ^5^** | **Efficiency^6^** | **Relative mRNA abundance^7^** | **1/E∆Ct^8^** | **%** |
| --- | --- | --- | --- | --- | --- | --- | --- | --- | --- | --- |
|  | |  |  |  |  |  |  |  |  |  |
| ***PPARG*** | 29.375 | | 6.339 | -2.459 | 0.999 | 155.07 | 2.550 | 0.003 | 0.001 | 0.084 |
| ***LPL*** | 23.804 | | 0.690 | -2.513 | 0.999 | 150.78 | 2.500 | 0.531 | 0.170 | 16.95 |
| ***CEBPG*** | 25.300 | | 2.341 | -2.548 | 0.999 | 146.90 | 2.469 | 0.121 | 0.038 | 3.848 |
| ***CEBPD*** | 23.269 | | 0.183 | -2.610 | 0.999 | 141.73 | 2.416 | 0.851 | 0.272 | 27.16 |
| ***DNMT1*** | 26.948 | | 4.044 | -2.318 | 0.996 | 171.75 | 2.700 | 0.018 | 0.006 | 0.57 |
| ***SOD2*** | 22.668 | | -0.509 | -2.568 | 0.999 | 145.29 | 2.452 | 1.579 | 0.504 | 50.39 |
| ***NOS3*** | 26.793 | | 3.627 | -2.407 | 0.998 | 160.45 | 2.603 | 0.031 | 0.010 | 0.99 |
|  |  | |  |  |  |  |  |  |  |  |

1-The median is calculated considering all time points and all calves.

2-The median of ΔCt is calculated as [Ct gene – geometrical mean of Ct internal controls] for each time point and each calve.

3-Slope of the standard curve.

4-R^2^ stands for the coefficient of determination of the standard curve.

5-Efficiency (%) is calculated as [10(-1 / Slope)]-1 x 100.

6-Efficiency is calculated as [10(-1 / Slope)].

7-Relative mRNA abundance = 1/ Efficiency Median ΔCt.

8-1/EΔCt = relative mRNA abundance/Σ relative mRNA abundance
